# Use of probabilistic fastener velcro as a friction-induced vibration damping treatment

**DOI:** 10.1038/s41598-022-23946-8

**Published:** 2022-11-12

**Authors:** Semin Kwon, Jonghoon Jeon, Seungjeok Yoo, Junhong Park

**Affiliations:** Department of Mechanical Engineering, Hanyang University, 222 Wangsimni-Ro, Seongdong-Gu, Seoul, 04763 Republic of Korea

**Keywords:** Engineering, Mechanical engineering

## Abstract

Probabilistic fasteners are biologically inspired clamping devices that are interlocked by stems on each surface. Due to dynamic characteristics of fastening mechanism, friction inevitably occurs between stems in a vibrating environment. The use of the probabilistic fastener as a vibration reduction component were investigated with advantages from friction-induced damping in this study. The dynamic stiffness and loss factor of the probabilistic fastener were derived from the vibration interaction with a mechanical structure. This allowed determination of energy dissipation due to the friction in hook and loop from the wave propagation analysis. As the vibration amplitude increased, the loss factor of the fastener gradually increased because the friction between multiple stems increased. With the probabilistic fastener application, the vibration generation and transmission were reduced compared to the bolted joint due to the inherent frictional contacts. With this unique advantage, the probabilistic fastener has potential applications when large damping is required with additional benefit on the reduced weight.

## Introduction

The demands for noise and vibration reduction are increasing for improved performance of mechanical systems such as electronic appliances and automobiles. Reduction of unnecessary vibration is a significant design factor for verification of durability, accuracy, and quality performance of a machine system. Vibration energy is transmitted from operating components including motors, engines and compressors through fasteners to surrounding environments. Fasteners in assembled structures reduces the vibration transmission and prevent buzz, squeak, and rattle (BSR) noise. The BSR noise generates by frictional contacts between adjacent components^1^. The flexural wave energy transmission analysis is required to analyze the influence of the joints on the vibration behavior^2^. The wave approach considered several combinations of rib depth and bolt spacing. In-vehicle noise was measured when trains were running at different speeds over the same non-ballasted track section equipped with two types of rail fasteners^3^. According to the stiffness of the rail fasteners, the spectra of the internal noise showed the frequency band of air-borne and structural-borne noise generations. In order to prevent the BSR noise, several effective design practices were proposed^4^. The fasteners along the trim surface and hybrid fastening systems that incorporated both load and non-load bearing joints were recommended.

Threaded bolts are widely used due to several advantages such as simple operation, low cost, and high tensile strength. The bolt fasteners lose their clamping force by self-loosening especially when exposed to transverse excitations^5^. The bolt loosening induces failure of jointed connections. It is important to increase the bolt reliability by prevention of self-loosening^6^. Toh et al.^7^ evaluated the clamping force of the bolt in the vehicle lower arm through the vibration resonance frequency. The dynamic stiffness of bolted joint was evaluated in laminated composites by the flexural wave propagation analysis^8^. In order to increase the vibration reduction performance of bolted joints, viscoelastic damping material were used^9^.

Recently, the conventional fastening methods have been replaced by adhesives. Adhesive bonding has the advantage on minimal distortion of the component compared to the welded parts^10^. The adhesive bonding provides improved stiffness compared to conventional fasteners or spot welding because it creates continuous bond rather than local point contacts. As a result, stresses evenly distributed over a large area were generated in the fastener^11^. The adhesive exhibited good energy absorption performance and induced efficient noise and vibration damping properties^12^. With the advantages on application to composite structures, adhesive bonding applications are expanding rapidly. Adhesive bonding has limited application for systems requiring reassembly.

**Figure 1:**
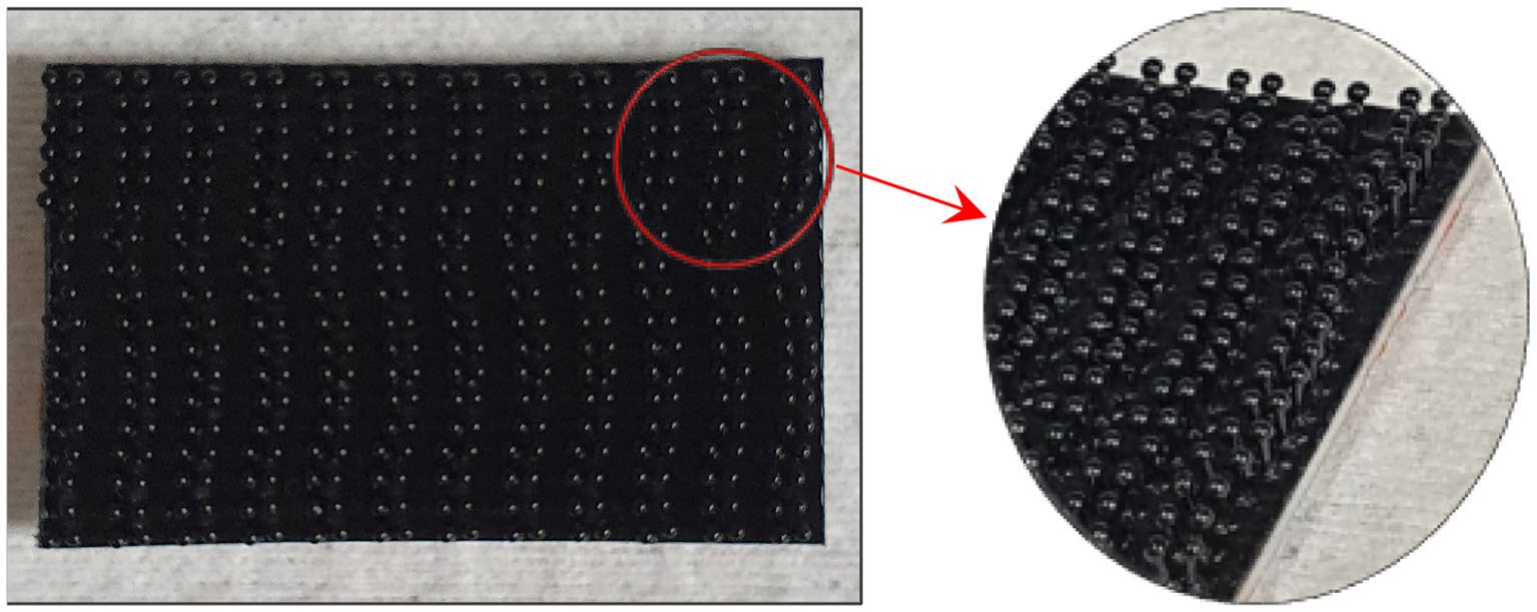
Dual-Lock fastener consisting of polyolefin backing covered with small mushroom-shaped stems.

Velcro® is a hook-and-loop type of probabilistic fastener inspired by a variety of nature, including wood, burdock seed and spider leg^13^. With the increasing demands of fastening devices, various types of novel probabilistic fasteners were proposed^14^. As shown in Fig. 1, the Dual-Lock® fastening system consists of a polyolefin backing covered with small mushroom-shaped stems. Due to the impact-resistant joining performance from flexible materials, this probabilistic fastening system is used in variety of joint applications in the automotive industry, including trim strips, headliners, sunroof and door panels. The noise of vacuum-assisted devices was reduced by using Velcro® as a layer damping material^15^.

The hyper-elastic nonlinear behavior of fasteners was measured as a function of the friction and displacements^16^. In the shear direction, the friction dynamics of hook-and-loop systems were explored according to typical parameters of drive speed, applied load, and apparent contact area^17^. A study on the peel tests were conducted through the behavioral characteristics of the hook and loop bond mechanism^18^. Recently, a novel hook and loop fastener made of thin nickel titanium wires was developed. The unique features of these fasteners, such as strength adjustment by thermal energy, reproducibility, and fastening robustness, were studied from the superelasticity of nickel titanium microwires^19^. Few studies have been conducted on the dynamic characteristics of probabilistic fasteners as vibration-reducing component. In order for the probabilistic fasteners to be applied to various operating systems, it is necessary to study the dynamic characteristics. Chowdhury et al.^20^ investigated the effect on the friction coefficient according to the amplitude of vibration for various materials. As the amplitude of vibration increased, the friction coefficient of the material decreased, which means that the ability of material to dissipate vibration energy into other energy such as thermal energy decreased. Since the stems constituting the probabilistic fastener are entangled and held together, contacts occur due to tension and compression behavior in a vibration environment. The relative motion of two objects induces a local damping from friction caused by vibrating contact. The forced response of a vibroimpact system is analyzed as follows^21^:

1 $$m\ddot{u} + (\lambda u^{n} )\dot{u} + ku^{n} = 0,$$

where *u* is the displacement toward the moving direction, *m* is the mass of the moving body, *k* is the linear spring constant, *n* is the value for Hertzian elastic point contact, and $\lambda$ is the damping factor. The damping factor is calculated as follow:

2 $$\lambda = 1.5\alpha k,$$

where *α* is the coefficient of restitution. To characterize the frictional characteristics, it is necessary to evaluate the damping properties and effects of the hysteresis loop for the multiple stems in the probabilistic fastener.

In this study, experiments were conducted to understand a vibration reducing capability by the frictional properties of probabilistic fastener. In order to measure the dynamic properties, a vibration test was performed for the cantilever beam interacting with the fastener support. The influence of fastener was analyzed by the translational stiffness. The vibration interaction was analyzed and compared with the theoretical model for evaluations. The influence of the elongation thickness of the stems on the fastener behavior was studied. The vibration damping performance of the probabilistic fastener was also investigated in comparison with polymer materials. The influence of the friction caused by vibration occurring in the fastener on the vibration reduction performance was measured. Vibration tests were performed using the two identical beams jointed by the probabilistic fastener. The fastening performance was compared to those of the bolted joint.

## Method

### Wave propagation model

The fastener properties influence on the vibration of the supporting structure. To analyze the vibration interaction, the supported structure vibration was analyzed as

3 $$D\frac{{\partial^{4} w}}{{\partial x^{4} }} + M_{b} \frac{{\partial^{2} w}}{{\partial t^{2} }} = 0$$

where *w* is the transvers displacement, *D* is the bending stiffness per unit length, and *M*_*b*_ is the mass per unit length^22^. For a harmonic vibration of $w(x,t) = {\text{Re}} \{ \hat{w}(x)e^{i\omega t} \}$, the vibration response is analyzed as

4 $$\hat{w}(x) = \hat{A}_{1} \sin \hat{k}_{b} x + \hat{A}_{2} \cos \hat{k}_{b} x + \hat{A}_{3} e^{{\hat{k}_{b} x}} + \hat{A}_{4} e^{{ - \hat{k}_{b} (x + a)}}$$

where $\hat{k}_{b}$ is the wavenumber, $\hat{A}_{i} \, (i = 1, \ldots , 4)$ are the complex amplitudes, respectively. The boundary conditions of the cantilever beam excited with a point force were given as

5a–h $$\begin{gathered} \hat{w}( - a) = \hat{w}^{\prime}( - a) = \hat{w}^{\prime\prime}(b) = \hat{D}\left( {\hat{w}^{\prime\prime\prime}(0^{ + } ) - \hat{w}^{\prime\prime\prime}(0^{ - } )} \right) + \hat{S}_{t} \hat{w}(0^{ + } ) = 0,\,\, \hfill \\ \hat{w}^{\prime}(0^{ - } ) = \hat{w}^{\prime}(0^{ + } ),\,\,\,\hat{w}^{\prime\prime}(0^{ - } ) = \hat{w}^{\prime\prime}(0^{ + } ),\,\,\hat{D}\hat{w}^{\prime\prime\prime}(b) = F,\,\,\,\hat{w}(0^{ - } ) = \hat{w}(0^{ + } ) \hfill \\ \end{gathered}$$

where *F* is the applied force at the free-end, $\hat{D} = D(1 + j\eta_{D} )$ is the complex bending stiffness, $\hat{S}_{t} = S_{t} (1 + j\eta_{{S_{t} }} )$ is the translational stiffness of the fastener, $\eta_{D}$ and $\eta_{{S_{t} }}$ are the corresponding loss factors, *a* and *b* are the lengths between fastener and both ends of the beam, respectively. In this study, the rotational stiffness of the fastener was neglected. Applying the eight boundary conditions of Eq. (5) to Eq. (4), the transfer function was obtained as

6 $$\Lambda e^{j\phi } = \hat{w}(x_{1} )/\hat{w}(b)$$

where *x*_1_ is the location of the accelerometer installed on the beam, $\Lambda$ are $\phi$ the amplitude and phase of the transfer function. The vibration properties in Eq. (6) is the function of the Velcro complex stiffness, $\hat{S}_{t}$. The equation was solved using the Newton–Raphson method^23^. The complex stiffness obtained by the numerical method corresponded to the viscoelastic properties in the measured frequency bands.

### Experimental setup

**Figure 2:**
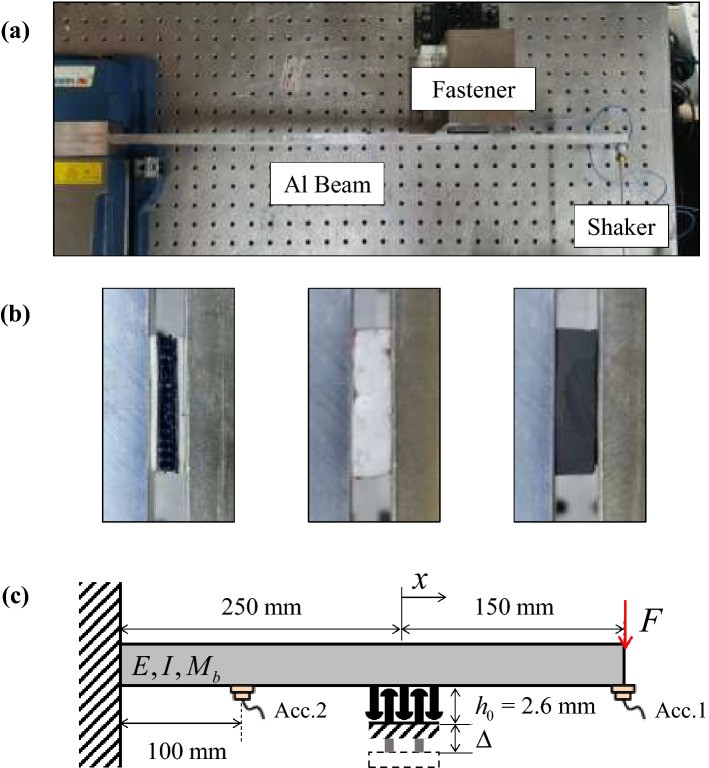
(**a**) Experimental setup of the cantilever beam attached with fastening specimen. (**b**) Probabilistic fastener, extruded polystyrene (EPS) and ethylene propylene diene monomer (EPDM), were attached to the beam. (**c**) Schematic of experimental setup to investigate the effects of the fastener thickness. Fastener and polymer materials were analyzed as the translational stiffness.

For evaluation of the damping properties of the Velcro joints, the vibration experiments were conducted at room temperature (21–23 °C). The experimental setup for the vibration test is shown in Fig. 2a. The aluminum beam was clamped at one end. The length, width and thickness of the beam were 400, 30 and 20 mm, respectively. A shaker at the free end provided the vibration excitation. The tests were performed with random excitation for 17.3 seconds. A total of 50 vibration responses were averaged to obtain the frequency response functions. The vibration responses of the beam were measured by accelerometers (Bruel and Kjaer, Type 4507) at 100 and 400 mm from the clamped end, respectively. The fastener specimen was installed in the direction of the excitation to support the beam at 250 mm from the clamped end. As shown in Fig. 2b, The probabilistic fastener specimen used in this study was Dual-lock® (3M, Type SJ3550). The extruded polystyrene (EPS) and ethylene propylene diene monomer (EPDM), which are widely used as polymer vibration damping treatments, were used in the experiments. The length and width of the probabilistic fastener and the polymer specimens were 20 and 30 mm, respectively. A summary of the experimental setup is presented in Table 1. In order to investigate the friction caused by vibration, the dynamic properties were measured with gradual elongation of the fastener until the detachment.

**Table 1:** Geometric parameters of the cantilever beam experimental setup.

	Length (mm)	Width (mm)	Thickness (mm)	Position (mm)
Al beam	400	30	20	–
Fastener	20	30	2.6 ~ 4.8	250
EPS, EPDM	20	30	2.6	250
Accelerometers	–	–	–	100, 400

Fig. 2c shows the schematic of the vibration experiments by which the dynamic properties were measured. The thickness of fastener is defined as follow:

7 $$h = h_{0} + \Delta ,$$

where *h*_0_ is the thickness when the fastener is most compressed, and $\Delta$ is the elongation length. The tests were performed varying the thickness of the fastener by 0.2 mm, from 2.6 mm (the fastener was at maximum compression) to 4.8 mm (the value prior to complete detachment). The spring element of the support has a significant influence on the vibration response of the structure^24^. In this study, the effect of the probabilistic fastener assumed as a translational spring at a single location was investigated through the wave propagation analysis of the vibrating beam.

**Figure 3:**
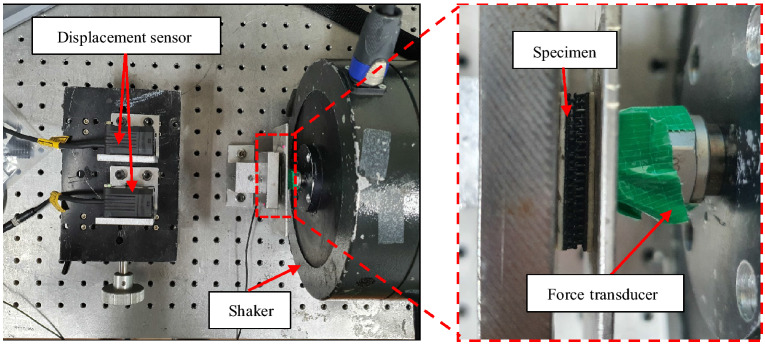
Uniaxial tension and compression test setup to measure the hysteresis loops of the polymer materials and the probabilistic fastener.

As shown in Fig. 3, a uniaxial vibration test was also performed to measure the hysteresis loops of the polymer materials and the probabilistic fastener. A flat plate for measuring displacement was inserted between the specimen and the force transducer. The other side of the specimen was attached to the fixed end. The length and width of the specimens of EPS, EPDM and probabilistic fastener were 25 mm and 20 mm, respectively. A force transducer was installed on the shaker to measure the force applied to the specimen. Two laser sensors were installed to measure the displacement of the plate that represented the specimen movement. While the specimen was excited at 100 Hz, the hysteresis loops were acquired from the force transducer and the displacement sensor.

## Results and discussion

### Dynamic properties according to elongation length

**Figure 4:**
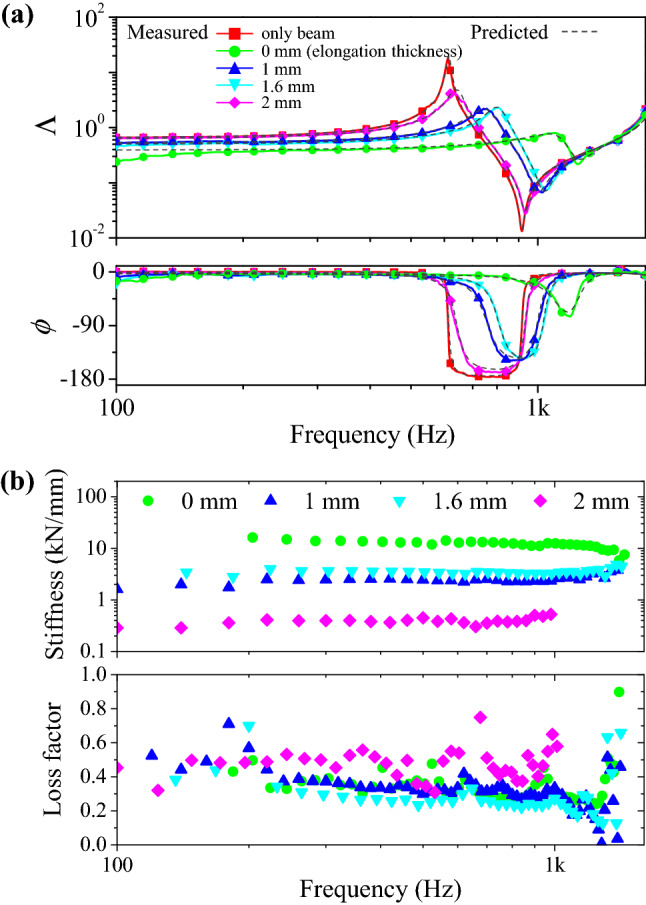
(**a**) Comparison of the predicted and measured vibration responses of the cantilever for different thicknesses of the probabilistic fastener. (**b**) The stiffness and loss factors of the probabilistic fastener calculated from the measured vibration responses.

Fig. 4a presents the vibration transfer functions of the cantilever with the probabilistic fastener. Figure 4 shows the functions for selected elongation thickness values (0, 1, 1.6 and 2 mm) from the performed tests. The measured vibration responses were compared and showed very close agreements with the predicted values using Eq. (6). For measurements without the probabilistic fastener, the damping was negligibly small. The probabilistic fastener increased the damping and the natural frequencies due to the support stiffness.

As the fastener thickness increased up to 1.0 mm, the resonant frequency decreased. Detachments of the stems from the fixed end induced the reduction of the fastener stiffness. As the thickness increased further than the values of 1.0 mm, the cantilever beam resonance frequency increased. This represented the increased fastener stiffness. The increase in the fastener stiffness continued until the thickness value was 1.6 mm. When the thickness increased further, the resonance frequency decreased due to stem separations and consequent detachments.

As shown in Fig. 4b, the dynamic properties of the probabilistic fastener were calculated from the measured vibration responses. At the maximum compressed status of the fastener, the stiffness was largest. As the elongation thickness increased to 1 mm, the stiffness became small. As the thickness increased further, the stiffness increased gradually. With the occurrence of partial separation with the increasing thickness to 2 mm, the stiffness became negligibly small. The loss factors were calculated to be 0.3 with minimal dependence on the compression status. The largest loss factor was obtained when the elongation thickness was 2 mm.

**Figure 5:**
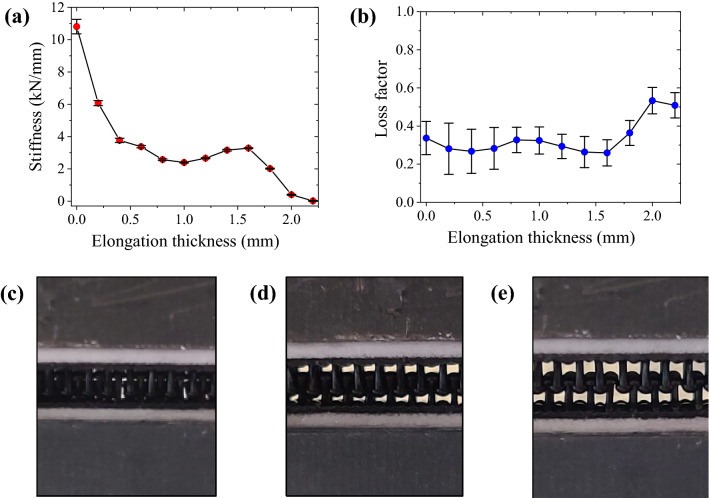
Dynamic properties of probabilistic fastener specimen, (**a**) stiffness and (**b**) loss factor. Specimen shape variations according to thickness variations: (**c**) 0 mm, (**d**) 1.0 mm, and (**e**) 1.6 mm.

The measured dynamic properties were averaged in the range of 100 to 1600 Hz, and the representative properties of the probabilistic fastener are shown in Fig. 5. Figures 5a and b show the influence of the fastener thickness on the average values and standard deviations of dynamic stiffness and loss factor. The results show close agreement with the one observed from the vibration resonance frequencies. Fig. 5c–e show the stems of the probabilistic fastener specimen. As shown in Fig. 5c and d, the deformed stems unfolded and the stiffness decreased sharply with the elongation of the fastener. The stiffness decreased in this range. In Fig. 5e, the heads of the probabilistic fasteners on the top and bottom interlocked with each other at the thickness of 1.8 mm and induced a slight increase in the stiffness. This local increase in stiffness was caused by the characteristics of the head and had the effect of preventing the probabilistic fasteners from separation^13,17^. With the further increase in the thickness, the separation of the heads occurred. As shown in Fig. 5b, the loss factors were measured as 0.3 for the specimen thickness of 1.6 mm. The loss factor increased to 0.6 from the thickness of 1.8 mm where the head separations occurred. When the probabilistic fastener was compressed or tensioned, it effectively suppressed the occurrence of relative contacts between the two structures with the increasing stiffness.

### Dynamic properties according to excitation magnitude

**Figure 6:**
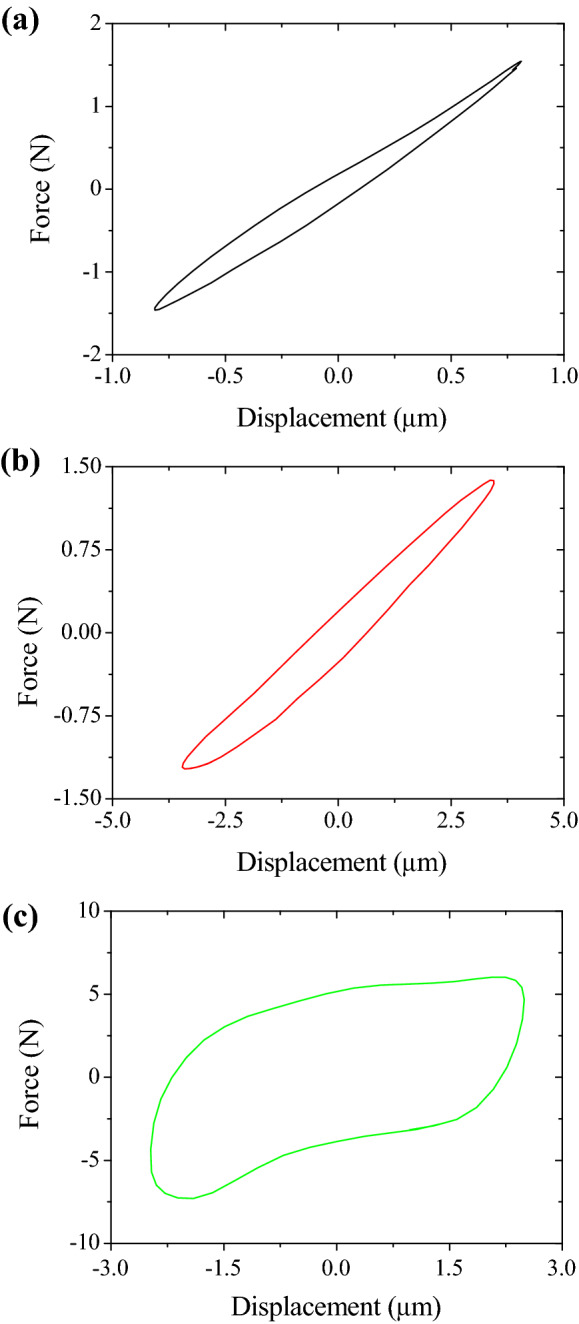
Hysteresis loops of (**a**) EPS, (**b**) EPDM, and (**c**) the probabilistic fastener.

For contacts between constituting components, the hysteresis loop induces the vibration energy dissipation^21^. The hysteresis loops of the polymer material and the probabilistic fastener generated in a vibrating environment is shown in Fig. 6. As shown in Fig. 6a and b, the hysteresis loops for EPS and EPDM behaved linearly for the small deformation occurred due to the external vibration. EPS and EPDM had almost constant stiffness regardless of the displacement. As shown in Fig. 6c, The stiffness of the probabilistic fastener changed depending on the displacement. When the displacement was large, the collision between the large stems increased the stiffness, which contributed to the increase in the area of the hysteresis loop. From the hysteresis loops, the damping ratio for EPDM, EPS and the probabilistic fastener were calculated as 0.087, 0.056, and 0.405, respectively. Since the probabilistic fastener is a structure in which heads are engaged, energy dissipation due to friction depended on the shape and property of the head. In addition, the increasing vibration amplitude caused greater frictional contacts between the heads, leading to a larger area of the hysteresis loop. The hysteresis loop represented the mechanical properties for a single frequency vibration. In this study, the beam vibration experiments were conducted to derive the mechanical properties of the polymer materials and probabilistic fasteners in the broad frequency band through the vibration transfer function.

**Figure 7:**
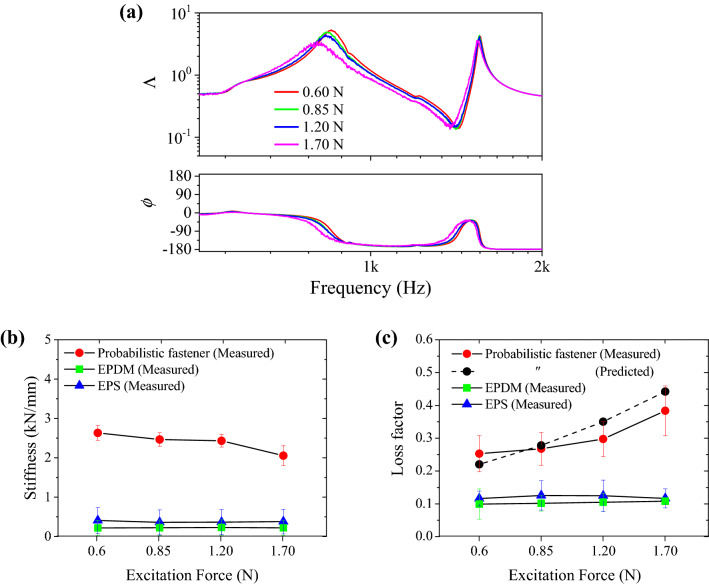
Effect of excitation magnitude on the vibration response with. (**a**) Measured vibration response of the cantilever supported with the probabilistic fastener. Comparison of dynamic properties of the probabilistic fastener and the polymer materials: (**b**) stiffness and (**c**) loss factors.

Vibrating stems in the probabilistic fastener undergo frictional contacts. In order to verify the damping performance by the contacts, vibration experiments were conducted with the increasing the response magnitudes. To control the magnitude of vibration, the input force of the shaker was increased from 0.60 to 1.70 N. To investigate the damping performance by the frictional contacts, the dynamic properties of probabilistic fastener and the polymer materials were compared. Figure 7a shows the vibration response of cantilever beam attached with the probabilistic fastener and the effects of the excitation magnitude. When the increasing excitation magnitude, the resonance frequency and vibration amplitude decreased. Figures 7b and c show the average dynamic properties and standard deviation obtained from the vibration tests. The viscoelastic properties of polymer materials were estimated constant regardless of the excitation intensity. The vibration damping performance of the polymer materials depended on the temperature^25^. In this study, we focused on the different damping mechanisms of the probabilistic fasteners from the polymer materials. Vibration tests were conducted at room temperature (21–23 °C). As shown in Fig. 7b, the stiffness of the probabilistic fastener was much larger than the polymer materials. As the excitation energy increased, the stiffness of the probabilistic fastener decreased. As shown in Fig. 7c, in the case of the smallest vibration magnitude, the loss factors of the probabilistic fastener, EPS, and EPDM were 0.253, 0.116, and 0.099, respectively. Moreover, in the case of the largest vibration magnitude, the loss factor of the probabilistic fastener increased to 0.384, while those for EPS and EPDM showed negligible variations as 0.117 and 0.108, respectively. The increasing damping performance of the fastener was much greater than the polymer materials in noisy environments. The predicted loss factor shown in Fig. 7c was derived by applying the properties of Dual-Lock, *n* = 1.2, and *α* = 0.2 s/m. The loss factor predicted by Eq. (1) also increased with the increasing vibration amplitude. As multiple stems entangled with each other, the hysteresis loop was generated, which exhibited excellent vibration energy dissipation performance. Through comparison with the polymer materials, the presented experiments showed the large vibration damping performance of the probabilistic fastener.

## Verification of damping performance of probabilistic fastener for jointed structure

**Figure 8:**
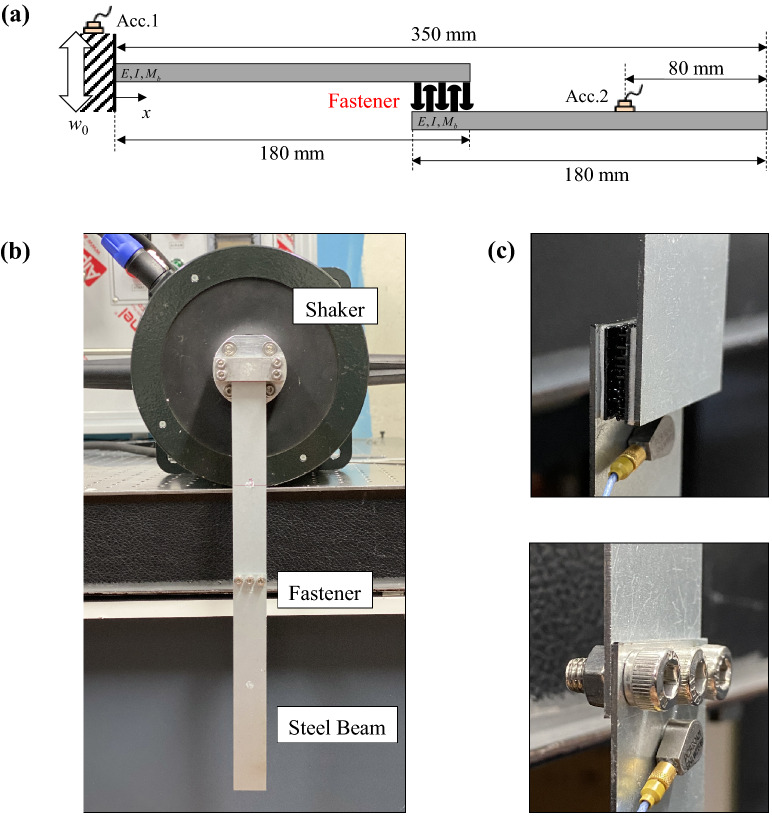
(**a**) Schematic of experimental setup (**b**) and its picture to investigate vibration transmission reduction performance of the fastener specimen. (**c**) Pictures of the probabilistic fastener and bolts connecting the beams.

In order to investigate the transmission reduction performance from the vibration source when applying the probabilistic fastener to the structure, a vibration test was performed on the joint structure with the setting as shown in Fig. 8. The vibration test was carried out by connecting two identical steel beams with fastener and clamping one end to a vibration shaker. As in the previous experiments, the shaker provided the random excitation for 17.3 seconds. A total of 50 vibration responses were averaged to obtain the vibration responses. The length, width and thickness of the beam were 180, 30, and 1 mm, respectively. The vibration responses of the beam were measured by accelerometers at 0 and 270 mm from the clamped end, respectively. As shown in Fig. 8c, the same Dual-Lock as in the previous experiment was used to fasten the two beams. For comparison of the vibration transfer performance, three conventional stainless-steel bolts and nuts were used to fasten the two beams at the same location. The diameter of bolt was 5 mm. The bolt pitch was 0.8 mm.

**Figure 9:**
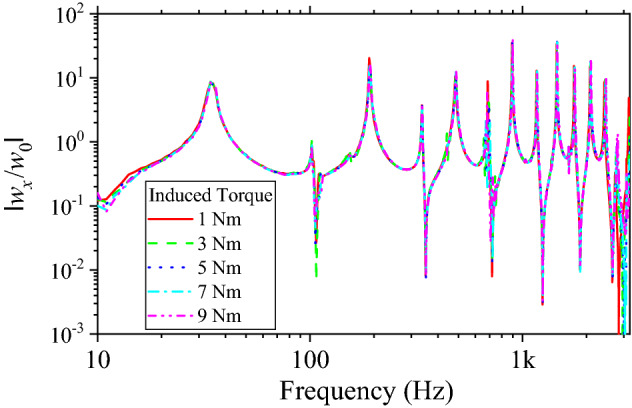
Vibration response of beam according to bolts tightening torque.

The vibration response of structure was affected by the bolt clamping force. However, the range of excitation used in this study is up to 3200 Hz, so the effect of the change in vibration response according to the fastening condition of the bolt was negligible except for very extreme situations. The high-frequency mode was affected only when the bolt tightening is completely loose. Figure 9 shows the vibration response for variation of the bolt tightening torque. The bolts were tightened using a torque wrench (Tohnichi). When the tightening torque of the bolt was larger than 2 Nm, the bending vibration response of the beam structure did not showed variations. The tightening torque of 7 Nm was used as a representative value and used for comparison.

**Figure 10:**
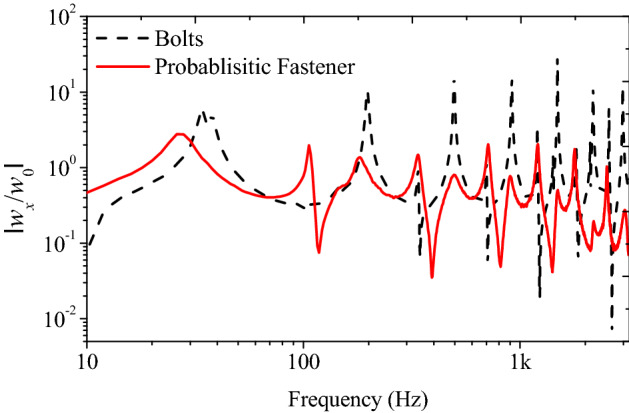
Vibration response of beam structure assembled with different fasteners – Probabilistic faster and bolt.

The vibration transfer functions are shown in Fig. 10. The weight of the Dual-lock used in the experiment was 0.98 g. The bolt and nut were heavier as 2.18 g and 1.23 g, respectively. Because the probabilistic fastener showed lower dynamic stiffness than the bolted joint, the measured vibration resonance frequencies of the jointed beams were smaller. As a result of calculating the Q-factor for the first peak, 3.34 and 11.41 were obtained for bolts and probabilistic fastener, respectively. Because the probabilistic fastener has high damping performance due to friction, the magnitude of vibration at the connected beam was significantly reduced compared to those assembled by the bolted joint. For the vibration magnitude from 10 to 3200 Hz, the probabilistic fastener showed a 6.1 dB reduction in the vibration transmission compared to the bolted joint.

## Conclusion

Probabilistic fastener is a clamping device interlocking onto their respective surfaces, providing high energy dissipation by friction in a vibrating environment. A study was conducted to investigate the dynamic characteristics of the probabilistic fastener as a vibration reduction component. Vibration tests were performed for different elongation thickness of the probabilistic fastener and the excitation magnitude. In this study, the effect of the fastener assumed as a translational spring at a single location on a vibrating beam was investigated through the wave propagation analysis. The complex stiffness in the frequency range was derived. The variation of the complex stiffness due to the stem contacting behavior was observed. The vibration damping of the probabilistic fastener was compared with the polymer materials. Since the friction and impact inside the fastener varied according to the vibration magnitude the vibration damping performance of the fastener also increased with the increasing magnitude of vibration. Vibration transmission was measured by connecting the two beams by the probabilistic fastener. When the probabilistic fastener was used instead of the bolted joint, the vibration reflection and transmission were reduced in the entire frequency band. These results suggest the advantage of the probabilistic fastener in prevention of unwanted vibrational contacts and responses of assembled structures.\

## Data Availability

All data generated or analyzed during this study are included in this published article.
